# Extracellular vesicles in pulmonary diseases: roles and therapeutic potential

**DOI:** 10.1186/s12890-025-03965-7

**Published:** 2025-11-10

**Authors:** Madison Coward-Smith, Razia Zakarya, Brian GG Oliver, Richard Y. Kim, Chantal Donovan

**Affiliations:** 1 School of Life Sciences, University of Technology Sydney, Sydney, NSW Australia; 2 Woolcock Institute of Medical Research, Macquarie University, Sydney, NSW Australia; 3 University of Newcastle, Hunter Medical Research Institute, Newcastle, NSW Australia

**Keywords:** Pulmonary disease, Infection, Extracellular vesicles, Immune modulation, Therapeutic opportunity

## Abstract

Extracellular vesicles (EVs) are small lipid bilayer packages responsible for cellular communication. Increasing clinical and experimental evidence strongly links EVs to homeostasis and the pathogenesis of disease. In this review, we provide a brief overview of EVs and their biological significance in pulmonary disease. We outline the current challenges in diagnosis and treatment of lung diseases and discuss the rationale for exploring EVs as a novel therapeutic avenue. Beyond their biomarker potential, we outline the role and potential for therapeutic targeting of EVs in the pathogenesis of asthma, chronic obstructive pulmonary disease (COPD), lung cancer, and infectious diseases. We also explore the current literature on the use of stem cell derived EVs to drive lung repair and regeneration in inflammatory diseases. Lastly, we highlight challenges and limitations of the study of EVs in pulmonary disease and provide future perspectives with exciting opportunities for translation into therapy.

## Introduction

###  Overview of extracellular vesicles (EVs)

**Fig. 1:**
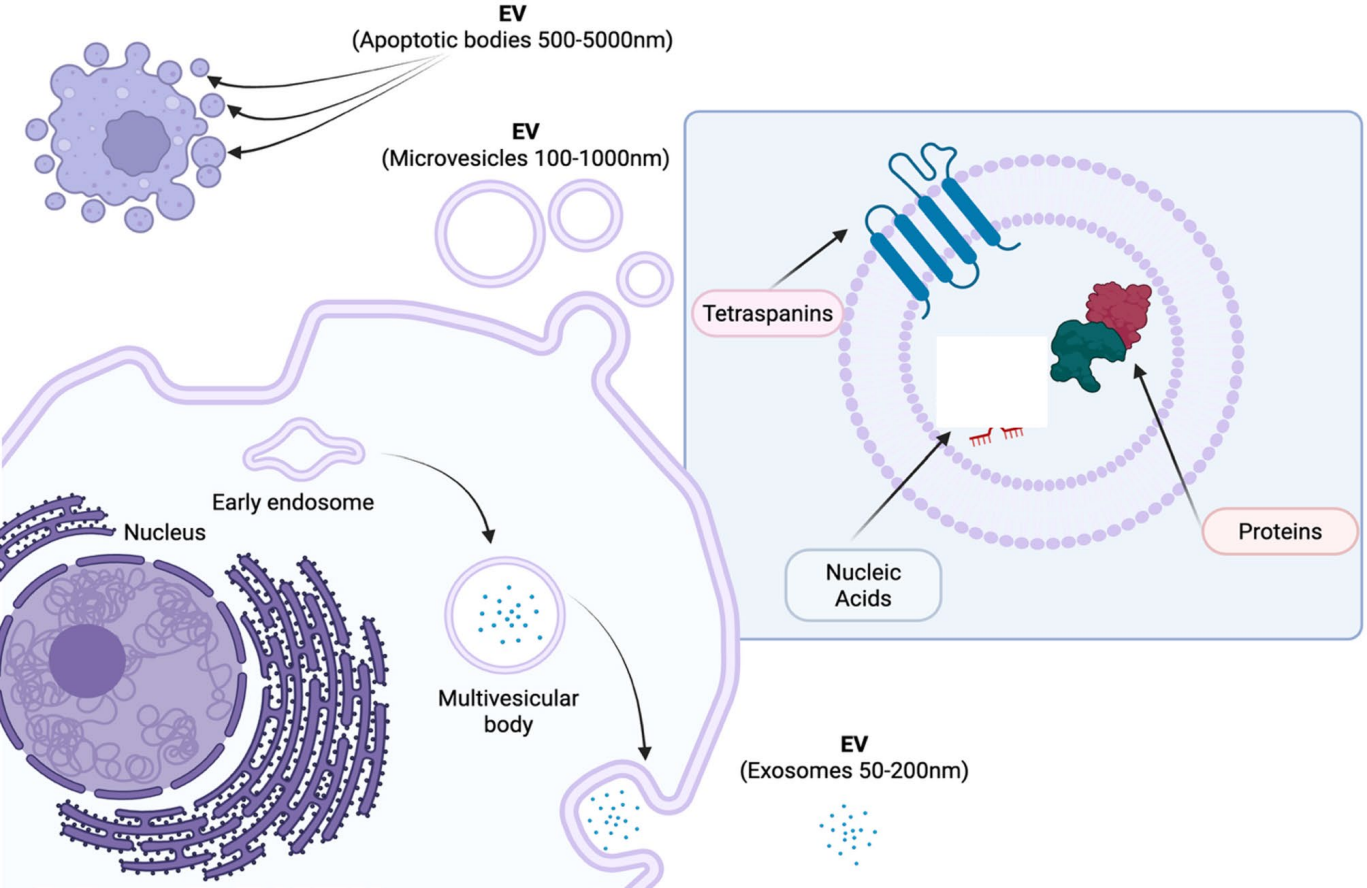
Extracellular vesicle biogenesis. *Created in BioRender. Zakarya*,* R. (2025)* https://BioRender.com/jdrn96u

Extracellular vesicles (EVs) are small lipid bilayer packages responsible for cellular communication. They contain an array of proteins, lipids and nucleic acids (miRNA, siRNA, lncRNA, RNA, DNA) [1] and play important roles in both physiological and pathological cellular processes [2]. Their biogenesis can occur through a variety of pathways such as exosomal transport (exosomes; 50–200 nm in size), membrane budding (microvesicles; 50–1000 nm in size) or other secretory pathways, such as from apoptotic cells (apoptotic bodies; 500–5000 nm) (Fig. 1). The minimal information for studies of EVs 2023 (MISEV2023) guidelines [3] suggest the general classification of EVs into either small EVs (sEVs; particles smaller than 200 nm) or large EVs (lEVs; particles larger than 200 nm) where it cannot be certain of the origin of the vesicle from the host cell. Throughout this review, the term ‘EVs’ will be used to describe exosomes, sEVs and lEVs. Traditional approaches for the collection of EVs include ultracentrifugation, size exclusion chromatography [4], precipitation and filtration, and may also involve the use of other methods such as microfluidic devices [5]. EV populations can be isolated from a range of biological fluids, such as breast milk or urine, as well as isolated from solid tissue samples and collected from cell culture supernatants. In this way, certain biological fluids may be useful for studying different diseases, such as urine for kidney function or bronchoalveolar lavage fluid (BALF) to investigate lung disease. Omics approaches, such as proteomics and transcriptomics have been invaluable in helping to understand the host cell and biogenesis pathways of EV populations, allowing for comprehensive profiling of EV cargo which can be further explored to gain functional insights of populations, as well as biomarker discovery [6, 7]. Collection of such biological fluids tends to be non- or minimally invasive, thereby placing great importance on such EV profiling studies.

Some of the more traditional approaches to the identification and purification of EV samples include nanoparticle tracking analysis (NTA), western blotting for EV enriched proteins and imaging through electron microscopy. However, rapid technological evolution within this nascent field has underpinned advancements in methods of isolation and characterization. These novel methods include the use of nano flowcytometry and super resolution microscopy, which provide highly detailed and descriptive analysis of single EVs [8].

###  Lung diseases: a global health challenge

Chronic lung diseases are a leading cause of death and disability worldwide [9]. These include asthma, chronic obstructive pulmonary disease (COPD), idiopathic pulmonary fibrosis (IPF), lung cancer, and infections. Together, they cause an enormous socioeconomic burden. Common features of these diseases include loss of lung function leading to severe breathing difficulties, acute and chronic airway inflammation and remodelling, and fibrosis. As a result, diagnosis and treatments for these diseases and infections are often ineffective, necessitating the need for better diagnostic tools and therapeutic options.

###  Rationale for studying EVs in pulmonary diseases

EVs play vital roles in cellular communication within the respiratory system. They contribute to the maintenance of lung homeostasis and can also play a role in disease progression and persistence [10]. For example, alveolar macrophages secrete vesicles which contains SOCS1 and SOCS3 protein which are taken up by alveolar epithelial cells to inhibit cytokine induced STAT activation both in vitro and in vivo [11]. Additionally, EVs collected from bronchial epithelial cells were able to attenuate Wnt signalling and subsequently myofibroblast differentiation and lung epithelial cellular senescence both in vitro and in vivo [12]. Conversely, EVs secreted from RSV infected cells show immune modulation in recipient cells (*in vitro)* by increasing the secretion of TNF, IP-10 and RANTES [13]. EVs are not only important in cellular signalling, but they also hold significant potential for biomarker discovery and as a result, may provide an avenue of the development of personalised medicine. This topic has been reviewed extensively elsewhere [14–17], and therefore falls outside the scope of this review. EVs have also been suggested as a potential therapeutic for regenerative medicine when isolated from mesenchymal stem cells (MSCs) in other models of disease such as osteoarthritis [18] and ischemic cardiomyopathy [19, 20], which would provide a promising clinical therapeutic avenue for currently irreversible lung diseases, such as COPD and IPF.

This review will focus on the current technology and future applications of EVs as therapeutic tools in respiratory diseases and infections. We will also discuss the current challenges in EV research, including issues with clinical translation. Finally, we will discuss the future of EV research and how EVs may be used in conjunction with existing medicine, or as new standalone therapies for respiratory disease.

## Role of EVs in pulmonary disease pathogenesis

###  EVs in inflammatory lung diseases

####  Asthma: role of EVs in immune modulation and airway inflammation

Increasing evidence implicates EVs in asthma pathogenesis and highlights their potential as biomarkers and therapeutic tools. Dendritic cells play crucial roles in asthma pathogenesis as the primary antigen presenting cell that captures, processes, and presents aeroallergens to T cells. Interestingly, dendritic cell-derived EVs can carry and present aeroallergens to induce the production of T2-associated cytokines clinically [21]. Other studies have shown that mast cells, which play central roles in allergic proinflammatory responses, can constitutively release EVs that are capable of activating T and B lymphocytes in vitro [22]. In contrast, others have shown that EVs derived from mouse bone marrow-derived mast cells (BMMCs) can affect allergic inflammatory responses by utilising FcεRI in their cargo to sequester free IgE, thereby decreasing IgE levels and inhibiting mast cell activation [23]. Eosinophilic accumulation in the lungs is a feature of allergic inflammation, denoting eosinophils as key effector cells in asthma pathogenesis and exacerbation [24]. Interestingly, there are reports that eosinophils isolated from patients with asthma produce more EVs than healthy subjects [25], and that eosinophils exposed to the eosinophil activators, CCL11 (eotaxin-1) and TNF, secrete more EVs [26]. There is also experimental evidence for the therapeutic potential of EVs in allergic asthma. De Castro et al.. assessed the effects of administering human adipose tissue-derived MSCs (AD-MSC) or EVs derived from AD-MSCs in a mouse model of allergic asthma [27]. The authors showed that systemic delivery of AD-MSC-derived EVs, but not AD-MSCs, reduces allergenic increases in static lung elastance and eosinophilic inflammation in the lungs. They also showed that treatment with AD-MSC-derived EVs decreases IL-4 and IL-5 levels in the lungs, but not IL-13 or eotaxin levels that were decreased by treatment with AD-MSCs, in experimental allergic asthma. Interestingly, both treatments decreased collagen deposition in the lungs, thereby highlighting the unique role that EVs play, separate from AD-MSCs. Taken together, these studies demonstrate the potential that underlies further characterisation of the disease-causing roles, biomarker, and therapeutic potential of EVs in asthma (Table 1).

####  COPD: EVs in chronic inflammation and tissue remodelling

COPD is also a chronic lung disease with hallmark features including inflammation and remodelling. Similarly to asthma, EVs in COPD are derived from various cell types and linked with disease pathogenesis (comprehensively reviewed in [28, 29]). Interestingly, numerous studies consistently report that the levels of EVs, particularly endothelial EVs, are increased in patients with COPD [28]. For example, there is a direct relationship between endothelial EVs and IL-6 responses, which suggests that proinflammatory signalling causes release of endothelial EVs [30]. The authors also demonstrated that the levels of monocyte-derived EVs are increased with increasing COPD severity [30]. Furthermore, microRNA cargo in EVs derived from bronchial epithelial cells is linked with airway fibrosis [31] and cigarette smoke-induced myofibroblast differentiation [32]. In addition to EV association with features of COPD, there is increasing evidence highlighting their potential utility as a diagnostic tool in COPD, including characterizing COPD phenotypes, distinguishing exacerbations from the stable state, and monitoring disease progression [28, 29]. Whilst EVs in COPD are a relatively new concept, lessons learned from EVs in asthma and other pulmonary diseases such as interstitial lung disease [33], idiopathic pulmonary fibrosis [34] and lung cancer [35] can be applied to COPD to advance this research.

###  EVs in lung cancer

Whilst pathologically different to asthma and COPD, lung cancer has been associated with chronic inflammation, and EVs have been shown to play key roles in the development and procession of lung cancer (comprehensively reviewed in[35–37]). The presence, and contents of, tumor-derived EVs can provide key information regarding cancer progression [38], stage/metastasis[39], and immune evasion [40]. To date, these EVs have been predominantly studied in liquid biopsies (e.g. blood). The cargo in these EVs can provide key information on cell growth, the tumor microenvironment, and invasive and metastatic activity of the cells that they are produced by. Whilst there has been a steady increase in the assessment of EVs in lung cancer, the maximum potential of these discoveries have yet to be fully explored and provides an important untapped opportunity.

In one study using EVs isolated from small cell lung cancer cell lines, it was demonstrated that different EV protein profiles are associated with drug resistance [41]. In a separate study, long RNA sequenced from EVs in serum of lung adenocarcinomas with tumour sizes < 2 cm showed considerable diagnostic value when used in conjunction with low dose CT scanning [42]. In that study, only long RNA samples were collected, isolation of EVs performed on fresh samples (not stored), and patients were recruited at later stage disease where they were admitted for surgical resections [42]. A separate study utilised EVs isolated from plasma in a microfluidic preparation, combined with machine learning to achieve 92.3% sensitivity and 100% specificity in distinguishing early-stage disease from benign lung disease [43]. These recent proof-of-principle studies highlight the innovation and novelty potential of EVs to predict lung cancer (Table 1).

**Table 1:** Summary of the role of EVs in respiratory inflammatory diseases

Pulmonary disease/condition	EV source	Type of study	Outputs	Therapeutic potential	Reference
Asthma	Mouse bone marrow derived mast cells (BMMC) P815 and MC9 mast cell lines	Mechanistic	Activation of T and B cells.	BMMC EVs are messengers and may be used to therapeutically alter the immune response during allergic response	^22^
Asthma	Mouse BMMC	Mechanistic	Reduced IgE.	BMMC EVs may be developed as a novel anti-IgE therapy	^23^
Asthma/Allergy	Human dendritic cells	Mechanistic	Present aeroallergens. Induce Th2-like cytokine production in allergic donors.	May be important targets in immunotherapy	^21^
Asthma	Eosinophils	Association	Increased eosinophil-derived EVs in asthma compared to healthy subjects.	Eosinophil EVs may be a therapeutic target in asthma	^25^
COPD	Endothelial cells in vitro and ex vivo patient samples	Association	Increased EVs compared to controls.		Systematic review outlining studies to date: ^28^
COPD	Blood samples from COPD patients: - Endothelial cell and monocyte-derived	Association	Positive correlation between endothelial EVs and plasma IL-6. Weak positive correlation between monocyte-derived EVs and IL-6.	Number of EVs may identify populations of high-risk patients for extra-pulmonary events	^30^
COPD	Bronchial epithelial cells	Mechanistic	MicroRNA cargo in EVs (miR-210) promotes myofibroblast differentiation in lung fibroblasts.	Bronchial epithelial cell EV miR-210 may be a therapeutic target for COPD	^31^
COPD	Bronchial epithelial cells	Mechanistic	MicroRNA cargo in EVs (miR-21) promotes myofibroblast differentiation in lung fibroblasts (MRC-5 cells).	Exosomal microRNA may be a biomarker of disease, and may be a treatable trait	^32^
Lung cancer	Blood (serum)	Diagnostic	23-gene long RNA signature distinguished EV samples of LUAD patients from benign controls.	Early detection of lung cancer	^42^
Lung cancer	Blood (plasma)	Diagnostic	EVs with lung cancer-associated markers PTX3 and THBS1 and canonical marker CD63, predicted early-stage lung cancer compared to benign lung disease and healthy controls.	Early detection of lung cancer	^43^

###  EVs in infectious lung diseases

EVs play important roles in the pathogenesis of respiratory infections not only via the trafficking of EVs from infected cells, but also through their dissemination into the blood stream, spreading inflammatory mediators throughout the body to induce the host immune response [44].

####  EVs in bacterial infections

Bacterial EVs are different to animal EVs in their biogenesis pathways, size, and composition with factors such as derivation from gram-negative or gram-positive bacteria affecting the EV. Bacterial EVs have been shown to contain chromosomal DNA, cell wall components, and toxins [45]. When EVs isolated from *Pseudomonas aeruginosa* were exposed to macrophages, macrophage changes in DNA methylation patterns occurred with subsequent changes in gene expression, which suggests an evasion strategy by the pathogen to alter the host immune response [46]. Furthermore, when *Pseudomonas aeruginosa* infected bronchial epithelial cell EVs were collected, they were found to be potent mediators of neutrophil migration, which may contribute to the excessive neutrophilic inflammation observed in these in vitro models [47]. Together these studies highlight the complex interplay between host derived and bacterial derived EVs and the immune response.

####  EVs in viral infections

EVs produced during viral infections have been reported to contribute to the cytokine storm. EVs from the serum of patients with SARS-CoV2 were found to contain elevated levels of IL-6, IL-2, IFN𝛾 and IL-2 when compared to EVs isolated from healthy controls [48]. Furthermore, EVs collected from the systemic circulation of patients with SARS-CoV2 and given intratracheally to mice, caused significant elevations in BAL cells and also increased pro-inflammatory cytokine concentrations [49] suggesting that systemic EVs contribute to lung inflammation.

EVs not only contribute to the pathogenesis of infection but have also been posed as new ways to boost the host immune response. For example, human bronchial epithelial cell EVs contain high levels of ⍺−2,3- and ⍺−2,6 linked sialic acid residues which can reduce H1N1 and H5N1 viral replication in vitro [50]. Furthermore, EVs have also been suggested as delivery vehicles for virus free vaccines, due to their ability to carry viral proteins to engage an antigen specific response [51–53]. This avenue of research presents a unique and promising opportunity for vaccine development and scientific discovery.

## Therapeutic potential of EVs in pulmonary diseases

###  EVs to target disease-associated features of pulmonary disease

EVs have been shown to interact with canonical and non-canonical pathophysical pathways. For example, wingless-type MMTV integration site family, member 3 A (Wnt3a) is an important component of the Wnt/β-catenin signaling pathway which is responsible for lung regeneration. In COPD, this pathway is dysregulated. The role of EV Wnt3a was explored in a study in which anchoring of Wnt3a to the surface of EVs was made possible by the transfection of HEK293T cells. EVs were then isolated from the conditioned media. Elastase-treated mice were prophylactically administered Wnt3a EVs or native EVs (2 × 10^9 particles per dose, intravenously) which improved lung function and reversed emphysema-like alveolar enlargement [54]. Thereby demonstrating how EVs can facilitate a physiological respiratory repair mechanism. Also, there are limited studies assessing the utility of EVs to reverse airway fibrosis. A single study showed that inhaled EVs from human lung spheroid cells (generated from whole lung samples) reversed mouse lung fibrosis induced by bleomycin or silica exposure [55]. EVs can also be used as drug delivery platforms, and anticancer vaccines in lung cancer (recently reviewed in ^56^).

####  Stem cell-derived EVs for lung repair and regeneration.

MSCs are multipotent stem cells which promote cellular differentiation proliferation and tissue regeneration. EVs produced by MSCs also share the same regenerative cargo as their parent cells, which makes them potential candidates for regenerative therapy [56].

Human MSC EVs can improve survival, mitigate lung inflammation, protein permeability, and bacterial growth in *Escherichia coli* pneumonia in mice [57], through leukotriene B4 signaling pathways [58]. A case report [59] found that the treatment of a pulmonary fibrosis patient with placental-derived MSC EVs showed that after several doses of EVs, there was a significant reduction in ground glass consolidations and fibrotic changes and a significant improvement in clinical symptoms at 12 months post treatment. However, there was no improvement in pulmonary function tests, and only involved one participant, highlighting the need for future development.

However, other types of stem and progenitor cells may also provide a valuable source of regenerative EVs. These may include basal cells, neuroepithelial bodies, bronchioalveolar stem cells and type II alveolar cells, all of which are capable of self-renewal in the lung [60]. Further research into the use of these EVs may prove vital in the creation of novel regenerative lung therapies.

###  Potential of EVs to modulate immune responses in inflammatory lung diseases

EVs can modulate the host inflammatory response in inflammatory lung diseases through the transfer of their cargo to various cells within the respiratory system. The majority of EVs produced in the respiratory system are derived from lung epithelial cells [61]. These EVs are bioactive, for instance, as EVs isolated from epithelial cells exposed to hyperoxia were found to activate macrophages and were rich in pro-inflammatory miRNAs [62]. Similarly, EVs from the BALF of mice who experienced a sterile or non-sterile respiratory insult, showed increases in the production of EVs, which promoted the recruitment of macrophages to the lung. These EVs also regulated the production of pro-inflammatory cytokines from alveolar macrophages [63], which highlights the potential of epithelial derived EVs to modulate lung inflammation. Not only do epithelial cell EVs communicate with macrophages, but studies have also shown that they also play an important role in allergic airway disease. Epithelial cells treated with IL-3 were found to have decreased expression of three miRNA’s which are involved in regulating Th2 differentiation and DC maturation [64], which may suggest a role for their involvement in the development of allergic airway disease. EVs from respiratory endothelial cells and platelets can also influence the development of inflammation within pulmonary vessels which may contribute to the development of vascular diseases such as pulmonary arterial hypertension (PAH). The presence of these EVs in the blood stream has been linked with the severity of PAH, with these EVs enriched in angiogenic proteins [65]. Together, these studies collectively highlight the role of EVs in the modulation of immune responses in inflammatory lung diseases (Table 2).

**Table 2:** Summary of the role of EVs in disease associated features of pulmonary disease

Pulmonary disease/condition	EVs used	Effects	Application	References
COPD	HEK293T- Wnt3a overexpressing EVs	Improved lung function and reduced emp	In vivo	^54^
Pulmonary fibrosis	EVs from human lung spheroids	Reduced airway fibrosis	In vivo	^55^
Bacterial pneumonia	Human MSC-EVs	Improved survival and mitigation of lung function	In vivo	^58^
Pulmonary fibrosis	Human MSC-EVs	Improved fibrotic changes, no improvement in lung function	Clinical	^60^
Allergic airway disease	Epithelial cell EVs	Decreased miRNA involved in Th2 differentiation and dendritic maturation	Human nasal lavage	^65^
Pulmonary arterial hypertension	Respiratory endothelial cell and platelet EVs	Influence pulmonary inflammation, contribute to disease development	Clinical	^66^

###  Current clinical trials involving EVs for respiratory disease

As of March 19, 2025, there are 25 registered clinical trials that involve the use of EVs and respiratory diseases. A phase III clinical trial (NCT05354141) is evaluating the use of bone marrow MSC-EVs to treat acute respiratory distress syndrome, and there are several other ongoing or completed clinical trials evaluating the safety and efficacy of MSC-EVs for COVID-19 infections and their associated symptoms (NCT 05787288, NCT04493242, NCT05808400). Other trials are analyzing proteins within circulating EVs to identify and validate biomarkers in small cell lung cancers (NCT05623956), and comparing EVs in cough samples, compared to bronchoscopy, for non-invasive biomarker discovery (NCT05854563). Collectively, these trials highlight the growing interest in the study and use of EVs in respiratory diseases and infections as both powerful tools for biomarker discovery and therapeutics.

## Challenges and future perspectives

### Challenges and limitations

Studying EVs in pulmonary diseases share similar challenges with all EV studies across all diseases. This includes challenges in EV characterisation, sample collection, analysis, yield, purity, contamination, storage and methodologies. Notwithstanding the MISEV guidelines establishing the minimum requirements, challenges with comparisons of pulmonary EV data across laboratories remain.

We have previously reviewed the general techniques, methodologies and challenges with scalability elsewhere. However, studying EV biology in lung diseases includes several organ-specific obstacles. These include contaminants due to the fact the lung is a mucosal surface that is constantly exposed to infectious, inflammatory, and noxious stimuli and as a result, lung fluids often contain numerous contaminants that can inadvertently be copurified with EVs. There are also limitations with the size of sample collections from patients, as these procedures are invasive, subject to variability, and can be associated with low EV yield.

Furthermore, the lung contains many constantly changing cell types that can dramatically increase the heterogeneity of EV populations, which can complicate defining the cell origin of the EVs. Together, this highlights the critical importance of following the MISEV guidelines and acknowledging the complexity of the sample collection in studies.

Adding to these challenges, the current literature on EVs comprises both in vitro and in vivo studies, which complicates the interpretation of the results. In vitro EV studies have several advantages, including often being cost effective, allowing for detailed analysis of a specific cell type, and in preclinical therapeutic testing. However, it is difficult to conduct long term models and predict the systemic effects of treatment. In contrast, while in vivo studies allow for the analysis of complex biological responses and longitudinal observations, they are often associated with biological variability and increased cost and may not be able to delineate the responses of specific cell types. Therefore, we propose that a combination of both in vitro and in vivo studies are required to advance the understanding of EV biology in pulmonary diseases.

### Considerations with translation into therapies

In pulmonary diseases, EVs have been shown to have pathogenic and protective roles. This may be due to the different cargo within the EVs, and/or the cellular source of the EVs in the lung. It is unlikely that pharmacologically inhibiting the release of pathogenic EVs from desired cells in the lung will have the specificity required for this to become a viable strategy to prevent or reverse pulmonary disease. For example, without specificity, there is the potential to also inhibit the release of beneficial EVs.

Potentially viable approaches are to intercept the EV/cargo delivering contents to recipient cells, or to neutralise the biological effects of the cargo pharmacologically.

In all cases, there are important considerations of the normal, homeostatic role that EVs play, including maintaining homeostasis and cell-to-cell communication. Modulating these in any way may alter immune responses and have potentially detrimental effects.

There is also potential of administering therapeutic EVs, which may include surface modification to increase targeting of cells to specific cell types, and/or with pharmacological interventions. For this to become a reality, we need to make several advancements in this field, including targeting specificity, maximizing bioavailability, minimizing adverse immune effects, and maximizing drug loading efficiency.

A further consideration is the need to define the specific EV subclasses with the most therapeutic potential. In reality, some subclasses may be more beneficial than others in different lung diseases, such as smaller EVs for delivery to tumours and larger EVs for drug-loaded delivery [66]. Furthermore, the mechanisms of EV uptake, especially in various lung cells, is an important knowledge gap in pulmonary EV biology, which will be critical for the translation of EV and EV-modifying therapies for lung diseases.

## Conclusion

Over the past decade, there have been significant advances in the understanding of EV biology in lung diseases. Importantly, this opens an exciting new avenue to transform treatments for different lung diseases. We identify key regulatory limitations and challenges that need to be overcome, including the standardization and rigor of reporting on EVs, which are on course to be improved with the MISEV23 guidelines. Furthermore, the identification of the appropriate host and size of the EV, and considerations as to whether EVs should be cell-derived, engineered, or synthetically generated for specific applications, will guide the future of EV-based pulmonary research. We propose that all EV sources hold potential for lung research in the future and specifically identify cell-derived EVs for their promising potential in the treatment of fibrotic lung diseases, and the application of engineered or synthetic EVs to improve drug delivery.

Further considerations should also be made to improve the scalability of EV production as current isolation methods are often time consuming, require specialised equipment, and may not generate clinically relevant therapeutic doses. These obstacles currently limit their capacity for clinical translation but will be overcome by advances in manufacturing and bioreactor-based production of EVs.

The immense potential for biomarker discovery and the therapeutic advantages of using EVs for the treatment of pulmonary diseases engender an appealing avenue for further research and clinical validation. Importantly, these studies must carefully consider the requirements of reporting on EV methodology, scalability of production, and storage and transport. Collectively, the rapid increase and uptake of the aforementioned technological advancements optimally position EVs for the transformation of understanding and treating pulmonary diseases.

## Data Availability

No datasets were generated or analysed during the current study.
